# Computer-Assisted Instrument Guidance to Improve Adductor Canal Block Performance for Total Knee Arthroplasty: A Pilot Randomized Controlled Trial

**DOI:** 10.7759/cureus.14300

**Published:** 2021-04-05

**Authors:** Ronak G Desai, Kiana D de Guzman, Noud van Helmond, Kinjal M Patel

**Affiliations:** 1 Anesthesiology, Cooper University Hospital, Camden, USA; 2 Anesthesiology, Cooper Medical School of Rowan University, Camden, USA

**Keywords:** regional anesthesia, adductor canal block, computer-assisted instrument guidance

## Abstract

Background

Postoperative pain associated with total knee arthroplasties (TKAs) is routinely managed with ultrasound-guided adductor canal blocks (ACBs). Computer-assisted instrument guidance (CAIG) systems can supplement the existing ultrasound machinery and block needles. CAIG systems allow the operator to navigate the needle in real time while displaying a projected trajectory of its path onto the ultrasound monitor. This study explored how ACBs performed with CAIG compare with conventional ultrasound-only ACBs in terms of block efficiency, success, and potential tissue damage for patients undergoing TKA.

Methodology

A total of 26 patients undergoing TKA under spinal anesthesia with an ACB were randomized to ACB utilizing conventional real-time ultrasound or to ACB utilizing real-time ultrasound supplemented with CAIG. The primary outcome measure was time to block completion. The secondary outcome measures included number of needle insertions, postoperative pain scores until postoperative day three, postoperative muscle weakness, opioid requirements on postoperative day zero, length of stay, and patient satisfaction with pain management.

Results

The time required to complete the block as well as the number of needle insertion attempts were similar between the CAIG and conventional ACB groups. Postoperative outcomes such as pain scores up to postoperative day three, postoperative muscle weakness, opioid requirements on postoperative day zero, length of stay, and patient satisfaction with perioperative pain management were comparable between the CAIG and conventional ACB groups.

Conclusions

CAIG does not reduce ACB performance times or patient outcomes when performed by experienced anesthesiologists.

## Introduction

In 2017, approximately 700,000 total knee arthroplasties (TKAs) were performed in the United States, and this number is predicted to increase to 3.5 million annually by the year 2030 [1]. Postoperative pain associated with TKAs is often intense and, when present, can prolong the hospital length of stay and recovery, as well as increase the risk of adverse events following surgery [1]. Patients undergoing orthopedic procedures routinely receive peripheral nerve blocks to manage postoperative pain [2]. In the context of TKA, clinicians often perform adductor canal blocks (ACBs) utilizing ultrasound-guided techniques to administer single-shot or continuous catheter regional anesthesia [3]. The process of precisely injecting local anesthetics into the adductor canal can be time consuming and painful for patients. Anesthesiologists are often under the pressure of time when performing these peripheral nerve blocks and must acquire specialized technical skills to perform them safely and effectively [4]. Additionally, the needle position is not always clear on traditional ultrasound [5], which may prolong the procedure time and may cause pain and discomfort if numerous needle passes are required.

Computer-assisted instrument guidance (CAIG) has been demonstrated to facilitate faster breast biopsy [6], and has been shown to improve accuracy, needle redirections, and goal performance metrics during needle placement training in gel models by resident physicians [7]. We hypothesized that peripheral nerve block success and patient experience (i.e., time and ease to complete the regional block) can be improved using CAIG.

The Clear Guide ONE (Clear Guide Medical, Baltimore, Maryland, USA) is a CAIG system that allows the operator to view the needle in three dimensions outside of the patient’s skin without having any part of the device come in contact with the patient’s body. This system uses proprietary algorithms to display a projected path and trajectory of the needle onto the ultrasound monitor [8]. Existing ultrasound machinery and needles are supplemented with this system, allowing operators to reference the conventional ultrasound image with the addition of a guide to the target nerve [8]. Using CAIG may be of benefit to the patient and clinician by reducing time and increasing the accuracy and efficacy of peripheral nerve blocks.

We studied the use of CAIG in the context of ACBs for TKA. Our primary hypothesis was that the addition of CAIG would reduce the overall time to complete ACB versus performing the ACB with conventional ultrasound-only technique. Secondary hypotheses included that decreased needle repositions and decreased needle punctures would lead to less tissue damage in CAIG ACBs compared to those without CAIG, as evidenced by lower postoperative pain scores, lower opioid requirements, and greater patient satisfaction.

## Materials and methods

Participants

We conducted a pilot randomized clinical trial to compare block performance times and postoperative pain with ACB using CAIG versus ACB performed without CAIG. This study was conducted at Cooper University Hospital in Camden, New Jersey between October 2015 and May 2016. Inclusion criteria included: 18 years of age or older, undergoing TKA under spinal anesthesia with a planned ACB for postoperative pain management, and ability to provide informed consent. Potential subjects were screened for enrollment through the institution’s operating room schedule and were informed of the possibility to participate by telephone one to three days prior to surgery. All subjects provided written informed consent after all study procedures were described in detail by an investigator. The Cooper University Hospital Institutional Review Board approved this study (IRB nr.: 15-095), and this study was registered on clinicaltrials.gov (NCT02614222).

Randomization and blinding procedures

Using a computer-generated random list, patients were assigned to either the control group, which received the ACB without CAIG, or the investigational group, which received ACB with the supplemental CAIG system. Patients receiving the ACB and research staff who obtained postoperative information from patients were blinded to the assigned study arm, while clinicians performing the block could not be blinded.

Clear Guide ONE

**Figure 1:**
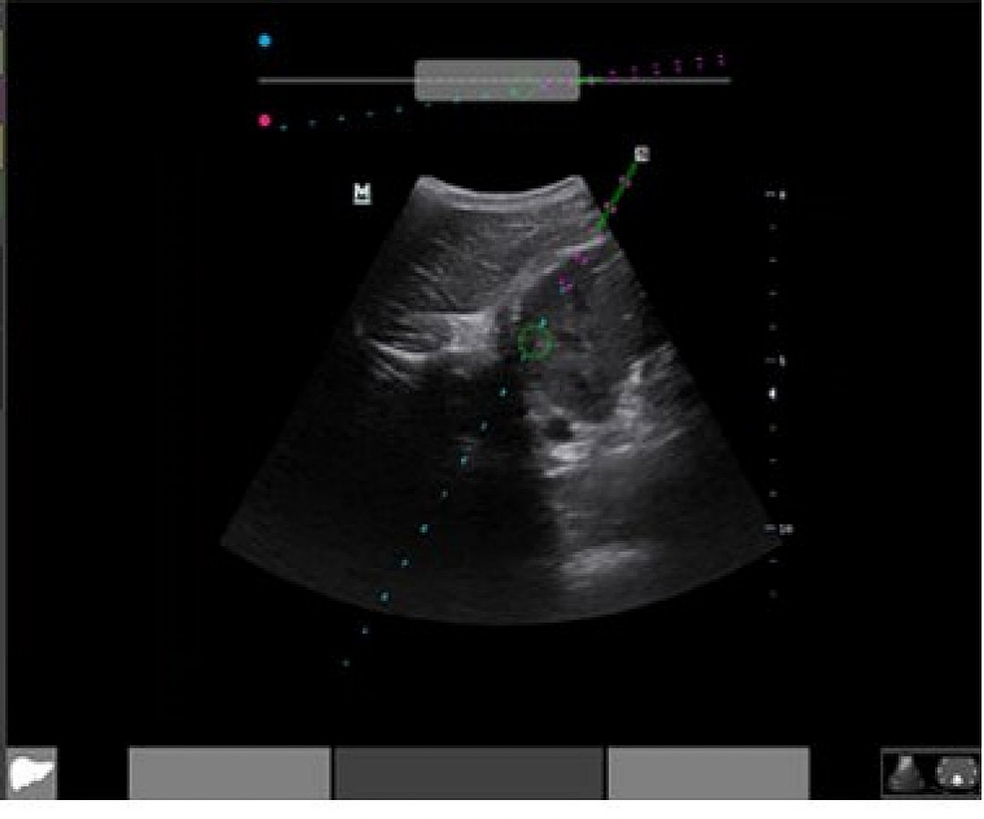
Exemplar ultrasound needle path projection with the Clear Guide system.

The Clear Guide ONE is a United States Food & Drug Administration-approved [9] system for augmenting the ultrasound visibility of interventional needles and for predicting the future needle path and trajectory. It is an optical system with stereo cameras that visualize the procedure needle in three dimensions prior to insertion. The Clear Guide CORE computer is connected to the ultrasound video output and the optical head is attached to a standard ultrasound probe. Proprietary algorithms calculate a projected path of the needle, which is overlaid onto the standard ultrasound image (Figure 1). The operator can confirm correct needle orientation prior to skin insertion and receive real-time feedback as the needle advances, which may aid in faster and correct needle placement.

Adductor canal block

The ACB was performed around the mid-thigh level after ultrasound identification of the femoral artery in the adductor canal. A 20-gauge, 100-mm echogenic needle (B Braun, Melsungen, Germany) was inserted in-plane in a lateral-to-medial orientation and advanced toward the femoral artery. Once the needle tip was visualized into the adductor canal and after careful aspiration, 1-2 mL of 0.25% bupivacaine was injected to confirm the proper injection site. When injection of local anesthetic did not appear to result in its correct spread circumferentially around the target nerve, additional needle repositions and reinjections were used until local anesthetic spread was optimized. After confirmation of correct positioning, the remaining volume of 0.25% bupivacaine was injected (20 mL total).

Three attending regional anesthesiologists performed all the adductor canal regional blocks in this study. Each anesthesiologist had a minimum of seven years post-residency experience and had performed more than 100 ACBs annually.

Anesthesia and postoperative care

All patients received spinal anesthesia using 2 mL of 0.75% bupivacaine with dextrose. Rescue opioid medication was administered when patients reported pain greater than 4 on a 0-10 numerical visual analog scale (VAS).

Outcomes

The primary outcome of this study was the time to ACB completion which was defined as the time from first ultrasound probe application on the operative limb to successful injection of all local anesthetics into the adductor canal, which was visually confirmed on ultrasound. Secondary outcomes included the number of needle insertions, opioid requirement on postoperative day zero, numerical VAS pain scores up to postoperative day three, a 0-10 patient satisfaction score with perioperative pain management, length of stay, and potential side effects of ACB, including but not limited to muscle weakness.

Data and statistical analysis

Total opioid medications administered throughout the hospitalization were converted to oral morphine equivalents using an opioid analgesic equivalent calculator [10] based on the American Pain Society guidelines [11] and several reviews regarding equianalgesic dosing [12-14]. Data were analyzed with SPSS software, version 24 (IBM Corp., Armonk, NY, USA) and visualized using SigmaPlot software, version 14 (Systat Software Inc, Chicago, IL, USA). Categorical data are presented as n (%), continuous variables are presented as mean ± standard deviation or median ± interquartile range (IQR), depending on the distribution of the data. Normality of the data was assessed using the Shapiro-Wilk test. Demographic data, treatment characteristics, and primary and secondary outcomes in the CAIG and conventional ACB groups were compared using unpaired t‐tests, Mann-Whitney U tests, or chi-square tests. Mixed linear model analysis was used to determine whether the use of CAIG resulted in differences in pain scores up to three days postoperatively. “Block Type” (CAIG/conventional) and “Time” (D0, D1, D2, and D3) were included as fixed factors. “Subject” was included as a random factor. To supplement this analysis, two‐sided post-hoc tests with Bonferroni correction were performed to determine at which time level differences existed if a significant main or interaction effect with P < 0.05 was detected for “Block Type.” P-values are reported for all testing.

## Results

**Figure 2:**
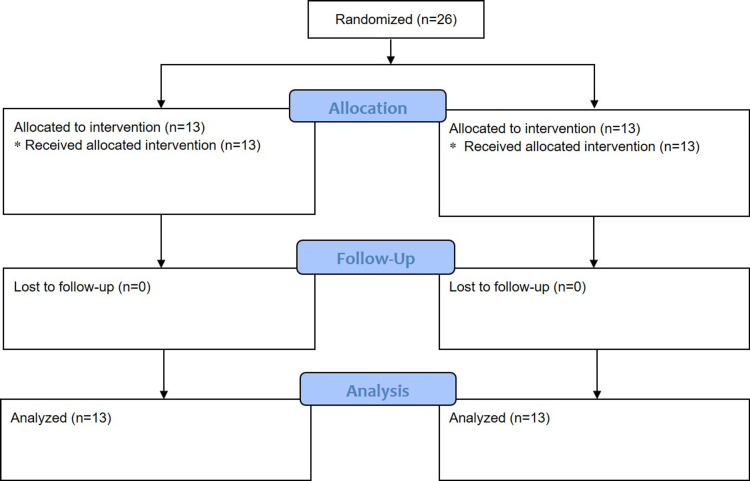
Flow chart of the trial.

A total of 26 patients were enrolled, randomized, and completed the study. The study flowchart [15] is presented in Figure 2.

Patient characteristics are displayed in Table 1. There were no significant differences between randomized groups.

**Table 1:** Characteristics of patients in the CAIG and conventional ACB groups. CAIG = computer-assisted instrument guidance; ACB = adductor canal block; TKA = total knee arthroplasty

Patient characteristics	CAIG ACB (n = 13)	Conventional ACB (n = 13)	P-Value
Age in years, median (IQR)	60 (50, 70)	61 (51, 71)	0.82
Sex, n M/F (%)	6/7 (46/54)	5/8 (38/62)	0.69
TKA side, n right/left (%)	5/8 (38/62)	8/5 (62/38)	0.24

Primary outcome

**Figure 3:**
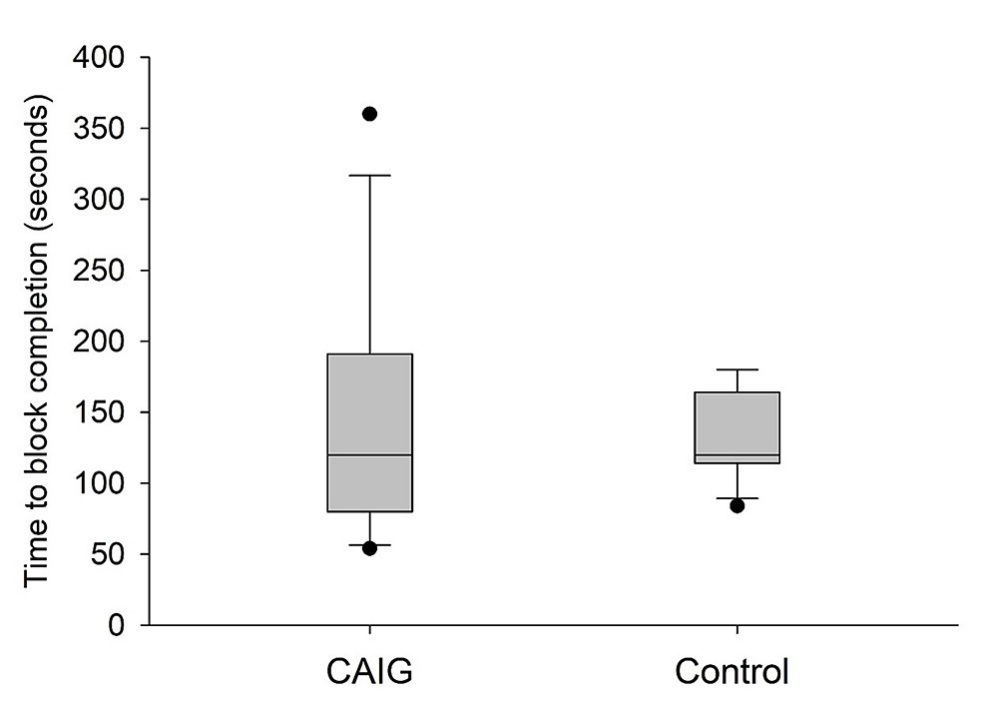
Duration of ACB procedure in the CAIG and conventional ACB groups. ACB = adductor canal block; CAIG = computer-assisted instrument guidance

Time to block completion was not statistically different between the CAIG (median = 120 seconds, IQR = 80-191 seconds) and conventional ACB (median = 120 seconds, IQR = 114-164 seconds) groups (Mann-Whitney U test; P = 0.76) (Figure 3).

The number of needle insertion attempts was similar between the groups (Table 2).

**Table 2:** Secondary ACB and postoperative study outcomes in the CAIG and conventional ACB groups. ACB = adductor canal block; CAIG = computer-assisted instrument guidance; IQR = interquartile range

	CAIG ACB (n = 13)	Conventional ACB (n = 13)	P-Value
ACB characteristics			
Needle insertion attempts mean (range)	1 (1-1)	1 (1-1)	1.0
Complications			
Muscle weakness, n (%)	2 (15)	1 (8)	0.54
Other, n (%)	0 (0)	0 (0)	1.0
Postoperative characteristics			
Opioid requirements on postoperative D0 in mg oral morphine equivalents, median (IQR)	60 (50–130)	45 (29–131)	0.14
Length of stay in days, median (IQR)	4 (3–4)	4 (3–4)	0.66
Satisfaction with pain management on 0-10 scale, median (IQR)	9 (8–10)	10 (9.25–10)	0.16

Secondary outcomes

**Figure 4:**
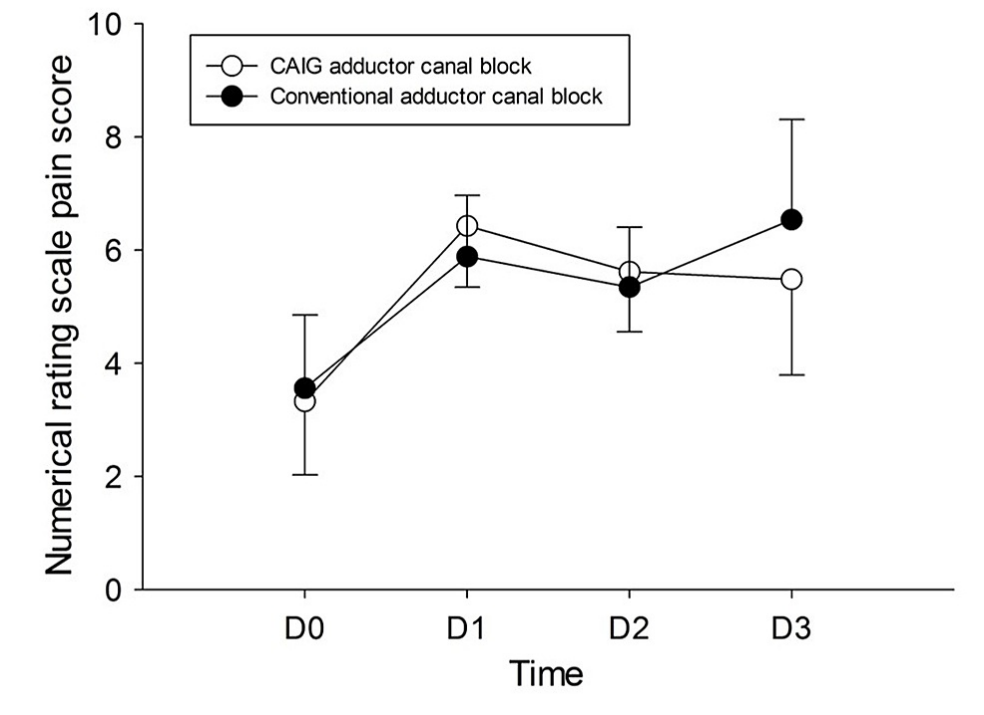
Pain up to postoperative day three in patients in the CAIG and conventional ACB groups. D0 = postoperative day zero; D1 = postoperative day one; D2 = postoperative day two; D3 = postoperative day three; ACB = adductor canal block; CAIG = computer-assisted instrument guidance

Pain scores up to three days after surgery were not statistically different between the study arms (Block Type P = 0.83, Block Type × Time P = 0.39; Figure 4). Additional postoperative outcomes including postoperative muscle weakness (P = 0.54), opioid requirements on postoperative day zero (P = 0.14), length of stay (P = 0.66), and patient satisfaction with perioperative pain management (P = 0.16) were comparable between groups (Table 2).

## Discussion

Principal findings

This pilot study explored the effect of using Clear Guide ONE, a CAIG system, on block performance and postoperative pain management in patients receiving ACBs. We predicted that compared to blocks performed without Clear Guide ONE those performed with CAIG would provide a 20% improvement in block efficiency and reduce postoperative pain scores, opioid consumption, and enhance patient satisfaction. We found that block performance characteristics were similar between the CAIG and conventional groups. Secondary outcomes of postoperative pain scores, opioid consumption, and patient satisfaction were similar between groups as well.

Previous studies on computer-assisted instrument guidance

Wilson et al. [7] studied the Clear Guide ONE system as an educational training tool in the context of ultrasound-guided needle placement in ballistic gel models by emergency medicine residents. They found that residents who performed needle placement with Clear Guide ONE had significant reductions in mean time to target and number of needle redirections and significant improvements in accuracy to target compared to those who did not perform with CAIG [7]. Of the training residents, 67% reported increased confidence with the technology and 95% perceived improvements in speed and/or accuracy [6].

Hauglum et al. [16] reported similar findings in their crossover study on the use of the Clear Guide ONE technology in simulated transverse abdominis plane (TAP) nerve blocks performed by student registered nurse anesthetists (SRNAs). The use of CAIG compared to ultrasound alone in TAP blocks resulted in reduced number of attempts and mean time to perform the block [16]. Compared to ultrasound alone, 68% of SRNAs perceived that blocks performed on porcine models with the CAIG system increased their confidence and ability to localize structures [16]. Surveys concluded that SRNAs had positive impressions and a preference for using the CAIG device over ultrasound alone [16].

Practical implications and future directions

The findings of the present pilot study do not support the use of CAIG for increasing the efficiency of ACB performance. Although the duration of block performance was similar between the CAIG and conventional ACB groups, it was striking that the conventional group had achieved block performance in a median time of 120 seconds. Moreover, all blocks were achieved in one attempt. These characteristics suggest that CAIG is of limited value in the context of improving performance when an ACB is performed by an experienced regional anesthesiologist in the clinical setting. It is possible that CAIG may have further value in more challenging blocks, or in blocks where needle and target visualization are problematic. Additionally, it is possible that this technology has added value in the context of learning regional anesthesia procedures in either the clinical or stimulated environment. Further studies involving the use of CAIG for training and education purposes in the realm of peripheral nerve blocks might provide additional information on the value of this technology in enhancing performance skills of newer regional anesthesia practitioners, potentially favorably influencing block efficacy and patient outcomes.

Limitations

This pilot study was limited in size and thus underpowered to detect small differences between the CAIG and conventional groups.

## Conclusions

CAIG does not reduce ACB performance times or patient outcomes when ACBs are performed by experienced anesthesiologists. Further studies involving the use of CAIG for training and education purposes in the realm of peripheral nerve blocks might provide additional information on the value of this technology in enhancing performance skills of more novice regional anesthesiologists.
